# Older People in Germany During the COVID-19 Pandemic:The Least, the More, and the Most Affected

**DOI:** 10.1007/s12062-021-09352-4

**Published:** 2021-12-13

**Authors:** Vincent Horn, Malte Semmler, Cornelia Schweppe

**Affiliations:** 1 Institute of Education, Johannes Gutenberg-University, Mainz, Germany; 2 Göttingen, Germany

**Keywords:** COVID-19, Pandemic, Older people, Latent class analysis

## Abstract

Older people have been identified as a particularly vulnerable group during the COVID-19 pandemic. However, the question of how older people actually fared during the COVID-19 pandemic has only been sporadically addressed. This article aims to partly fill this gap by classifying subgroups of older people using Latent Class Analysis. Indicators used are: risk perception, safety behavior, and well-being. To predict subgroup membership, age, gender, living arrangement, children, chronic illness, conflict, socioeconomic status, and migration history are controlled for. The data analyzed stem from a phone survey among 491 older people (75–100 years) in Germany conducted in September/October 2020. Results show that three subgroups of older people – the least, the more and the most affected – can be formed based on their risk perception, safety behavior, and well-being, indicating the usefulness of these three constructs for identifying and studying older people particularly affected by the COVID-19 pandemic and the measures taken to contain it.

**Supplementary Information:**

The online version contains supplementary material available at 10.1007/s12062-021-09352-4.

Since its outbreak in December 2019, the COVID-19 pandemic has caused the death of millions of people around the globe (John Hopkins University, 2021). Although individuals of all ages have died because of Sars-COV-2, there is little doubt that older people are at particular risk of severe coronavirus disease and mortality (Mueller et al., 2020; Sharma, 2021; Zhou et al., 2020). This also holds true for Germany, where in February 2021 the median age of people who had died with or because of Sars-COV-2 was 84 years (Robert Koch-Institut, 2021). In addition, a large proportion of the departed were multimorbid older people who lived in nursing homes (Robert Koch-Institut, 2020). Despite this finding, a strong tendency to homogenize older people can be found among policymakers and health authorities in Germany. As a consequence, all people aged 60 years and older are defined as in need of specific protection and especially called upon practicing social distancing and strictly following the hygiene recommendations (Bundesministerium für Gesundheit, 2020).

Like in Germany, governments in many other countries started campaigns and took preventive measures to protect older people, including social isolation, home confinement, and quarantine (Daoust, 2020). Researchers in the field of aging responded to these policies by pointing to the harmful consequences of social isolation and loneliness on older peoples’ mental and physical health (Brooke & Jackson, 2020; Hwang et al., 2020; Wu, 2020). In this regard, older people’s limited access and use of new communication technologies has been identified as a factor possibly exacerbating their social exclusion (Seifert et al., 2021). At the same time, researchers criticized using chronological age as the basis for defining individuals as particularly vulnerable and warned of the potentials of ageism as well as the possible fostering of paternalism due to the image of older people as uniformly weak and helpless (Ayalon et al., 2020; Petretto & Pili, 2020; Schulz-Nieswandt, 2020). However, the question of the extent to which the well-being of older people has been affected by the COVID-19 pandemic is difficult to answer.

Results from longitudinal studies investigating different dimensions of older people’s well-being before and after the outbreak of the COVID-19 pandemic are mixed. Studies from North America (Krendl & Perry, 2021; Luchetti et al., 2020), Norway (Hansen et al., 2021), and the Netherlands (Van Tilburg et al., 2020) found that loneliness among older people increased during the first wave. By contrast, studies on older people in Germany (Röhr et al., 2020) or Sweden (Kivi et al., 2021) did not detect any notable alterations in their mental health. Finally, exploring changes in loneliness among Spanish older people, Bartrés-Faz et al. (2021) found that ratings decreased shortly after the government had officially ordered home confinement. Another set of studies compared the well-being of older and younger people before and during the COVID-19 pandemic. Several of these studies found that older people are at a lower risk of psychological distress and loneliness than their younger counterparts (Birditt et al., 2021; García-Fernández et al., 2020; Glowacz and Schmits, 2020; Losada-Baltar et al., 2021). Such findings have been primarily explained by older people’s resilience and adaptive capacities to new life circumstances (Chen, 2020; Minahan et al., 2021; Röhr et al., 2020).

Older people, however, are not a homogenous group and therefore likely to dispose of different coping resources and strategies, health, social networks, living conditions, and so forth (Garcia et al., 2021; Gauthier et al., 2021; Tesch-Römer et al., 2020). Subsequently, while some older people may do relatively well (or even better), others may be worried about COVID-19 “to the extent that their well-being suffers” (Kivi et al., 2021: 7). There is indeed evidence of a close relationship between worry about COVID-19 and the behavior and well-being of people (Harper et al., 2020; Lanciano et al., 2020; Liu and Liu, 2020). People perceiving a higher risk of contracting COVID-19 disease, for instance, were found to be likely to show symptoms of anxiety as well as to adhere to quarantine guidelines (Carlucci et al., 2020). Similarly, studying the predictors of risk perception and older people’s behavior, Lu et al. (2021) found that higher risk perception is associated with a higher probability of preventive practices, including the avoidance of medical care. Different studies, however, indicate that risk perception and being worried decreases with increasing age, presumably because of a more relaxed attitude toward death (Bruine de Bruin, 2021; Guastafierro et al., 2021; Pasion et al., 2020). Especially, older men show relatively low levels of risk perception and are less prone to implement changes in their health behavior than women and younger people (Barber and Kim, 2021).

Methodologically, most studies conducted on older people in times of the COVID-19 pandemic used statistical procedures to explore correlations between different latent variables and covariates. However, thus far no study aimed to investigate different clusters or subgroups of older people based on the central constructs – risk perception, behavior, and well-being – identified in the literature. This study seeks to partly fill this gap by classifying qualitatively different subgroups of older people using latent class analysis (LCA). LCA uses the maximum likelihood principle to create internal homogeneous and externally heterogeneous subgroups (Weller et al., 2020). It provides information on the likelihood that an individual belongs to a specific class and allows extensions of the model by including covariates (Vermunt, 2010).

Starting from the theoretical premise that risk perception, behavior, and well-being are interrelated, all three constructs are included as indicators in our LCA model. In so doing, we aim to explore the extent to which the three constructs are suitable for identifying qualitatively distinguishable subgroups of older German people during the COVID-19 pandemic. Such knowledge would facilitate detecting older people who are particularly affected by the COVID-19 pandemic and developing social interventions specifically designed for them. Moreover, in order to predict subgroup membership, the association between subgroup membership and different covariates (age, gender, living arrangement, children, chronic illness, socioeconomic status, and migration history) is controlled for. The data analyzed stem from a phone survey among 500 older people (75–100 years) in Germany, which has been especially conducted to gain insights in their life situations during the COVID-19 pandemic.

## Data and Methods

### Data

The results presented in this study are based on data gathered in the context of the multi-topic survey of the forsa Institute for Social Research and Statistical Analysis. For this survey, 500 people aged 14 years and older are interviewed across Germany on a daily basis, using a multi-stage random selection system for telephone samples, which considers both landline and mobile phone numbers. Over a period of two weeks, people aged 75 and older participating in the survey were selected and asked separate questions designed by the author of this study. The procedure stopped when the previously defined sample size of 500 people had been reached. The aim of the separate questions was to gain information about changes in the lives and behavior of old and very old people during the COVID-19 pandemic, especially in relationship with a) their risk perception, b) safety behavior, c) social and emotional health, d) needs and support structures, and e) sociodemographic characteristics.

Data collection took place between September 28 and October 12, 2020. At that time, the first wave had passed, and previously taken measures to contain the pandemic had been relaxed. However, a slight but steady increase in COVID-19 infections in Germany could be observed, spurring a political debate about tightening measures again to contain a second wave of the pandemic. Compared to other European countries, such as Italy or Spain, Germany experienced a relatively light first wave with rather low numbers of infected and deceased citizens. In addition, although sporadically overloaded, there was no shortage of beds and ventilators in German intensive care units. All of this very likely influenced the older people’s perceptions of risk and behavior, and might also impact the responses given in this survey.

### Measures

We chose seven indicator variables to operationalize the constructs of risk perception, safety behavior and well-being (see Table 1 for how variables were coded). Two variables were chosen to measure the respondents’ *risk perception*: The degree of worry about getting infected with Sars-COV-2 and the anticipated severity of a possible infection. To capture their risk perception, respondents were first asked if they are currently extremely worried, very worried, not so worried, or not at all worried about contracting the COVID-19 disease. After this, they were asked to assess the consequences of a possible infection with Sars-COV-2 for themselves. Respondents could choose between five answer categories (very dangerous, rather dangerous, rather harmless, completely harmless, and don’t know). To reduce the number of categories, the answer categories “rather harmless” and “completely harmless” were collapsed into a single category.

**Table 1:** Descriptive Statistics of Variables

Variable		N	%
Risk perception			
Worried about infection	4: Not worried at all	99	19.8
	3: A little worried	241	48.2
	2: Very worried	140	28
	1: Extremely worried	20	4
Severity of infection	4: Don’t know	24	4.8
	3: Rather/completely harmless	90	18
	2: Rather dangerous	242	48.4
	1: Very dangerous	141	28.2
Safety behavior			
Avoids meeting family	2: No	411	82.2
	1: Yes	86	17.2
Avoids meeting friends	2: No	304	60.8
	1: Yes	195	39
Avoids public spaces	2: No	189	37.8
	1: Yes	310	62
Well-being			
Lacks social contact	3: Never	228	45.6
	2: Every now and then	169	33.8
	1: Often	103	20.6
Feels depressed	3: Never	377	75.4
	2: Every now and then	93	18.6
	1: Often	30	6
Covariates			
Age		500	80.46 (Mean)
Gender	1: Male	240	48
	2: Female	260	52
	3: Diverse	-	-
Living arrangement	1: Lives alone	227	45.4
	2: Lives with others	273	54.6
Child(ren)	1: Yes	442	88.58
	2: No	57	11.42
Chronic illness	1: Yes	241	48.39
	2: No	257	51.61
Education	1: Higher Education	222	44.4
	2: Lower Education	278	55.6
Property	1. Yes	341	68.2
	2. No	159	31.8
Income (household)	1: Low (0–2000 €)	146	29.2
	2: Medium (2001–3500 €)	165	33
	3: High (3501 € and more)	117	23.4
Migration history	1. Yes	56	11.2
	2. No	444	88.8

Three variables were chosen to measure the respondents’ *safety behavior*: Avoid meeting family members, avoid meeting friends and avoid public spaces. These three variables were part of an item battery designed to capture the impact of the COVID-19 pandemic on the social life of the respondents. Hence, the respondents were asked whether a statement (e.g., I avoid meetings with the family) fitted their current personal situation or not (yes or no). *Well-being* was measured by lacking social contact and moments of feeling depressed due to the onset of the COVID-19-pandemic. Lack of social contact was taken from an item battery on the respondents’ life situation as a whole. Respondents were asked how often (very often, now and then, never) something (e.g. the lack of exchange with other people) had happened since the beginning of the COVID-19 pandemic. The question of how often (very often, now and then, never) respondents experienced moments of feeling depressed was asked to gain information about the psychological consequences of the COVID-19 pandemic. While not reflecting the multiple dimensions of well-being, the variables refer to two dimensions (social and mental/emotional) which can be considered as particularly affected by the COVID-19 pandemic.

Respondents’ group membership was predicted by the following covariates: age, gender, living arrangement, children, chronic illness, socioeconomic status, and migration history. Age was a continuous and gender a categorical (male, female and diverse) variable. Living arrangement distinguished between those living alone and those who reported living at least with one other person in the household. Chronic illness was a dichotomous variable (yes or no) as well as having more frequent conflicts (e.g., discussions, getting on each other’s nerves) with family members since the beginning of the COVID-19 pandemic. Three variables were used to measure socioeconomic status: education (higher/academic vs. lower/non-academic education), household income (as measured in three income groups), and property (property vs. no property). Migration history was operationalized using the question of whether the respondent or at least one parent was born abroad (yes or no). Due to missing responses, the final sample consisted of 491 respondents. The relationship between the various variables was measured using Spearman’s coefficient for bivariate correlations (see supplementary material).

### Analysis

We used R 4.1.0 with the poLCA package (R Core Team, 2018; Linzer & Lewis, 2011) to conduct all analyses. We computed models with three, four and five latent classes or subgroups, respectively. According to goodness-of-fit measures, the models with two latent classes showed the best fit to our data (see supplementary material). Although the BIC was slightly more favorable in the case of the two-class model, we decided to opt for the model with three latent classes since it offers a more differentiated view at subgroups of older people in Germany during the COVID-19 pandemic. Latent class regression analysis was then used to control for the association between subgroup membership and the covariates age, gender, living arrangement, children, chronic illness, education, household income, property, and migration history. The covariates were calculated separately with the underlying base model, with an insignificant variation of percentages of the class membership of the three classes.

To test the robustness of our model, we randomly removed 30% of the surveyed subjects from the data set without a significant class change. The random removal was repeated 500 times, with deviations between ± 5% (Class 1) and ± 8% (Class 2). That class probabilities remain relatively stable, suggests that our model might be applicable to other data sets.

## Results

In this section, we first present findings from LCA in form of three different subgroups of older people and second, findings from exploring subgroup membership predictors, especially age, gender, living arrangement, children, and chronic illness.

How do older German people perceive and respond to the COVID-19 pandemic?

The majority of older German people was not very worried about getting infected with Sars-COV-2. More than two-thirds (68%) of the respondents reported to be less or not be worried at all. By contrast, 28% reported to be strongly and four percent to be extremely worried. In case of getting infected, however, most respondents anticipated severe consequences. Thus, more than 75% expected the consequences of a possible infection to be very or rather dangerous, compared to 18% who expected consequences to be rather or absolutely harmless.

As far as the respondent’s safety behavior was concerned, 17% reported avoiding meeting family members, 39% reported refraining from meeting with friends and two out of three (62%) stayed away from public spaces. More than half of the respondents reported that since the onset of the COVID-19 pandemic, they have missed social contact frequently (21%) or every now and then (34%). Six percent reported having experienced moments of feeling depressed more often since the onset of the COVID-19 pandemic, 19% reported passing through these moments every now and then.

**Fig. 1:**
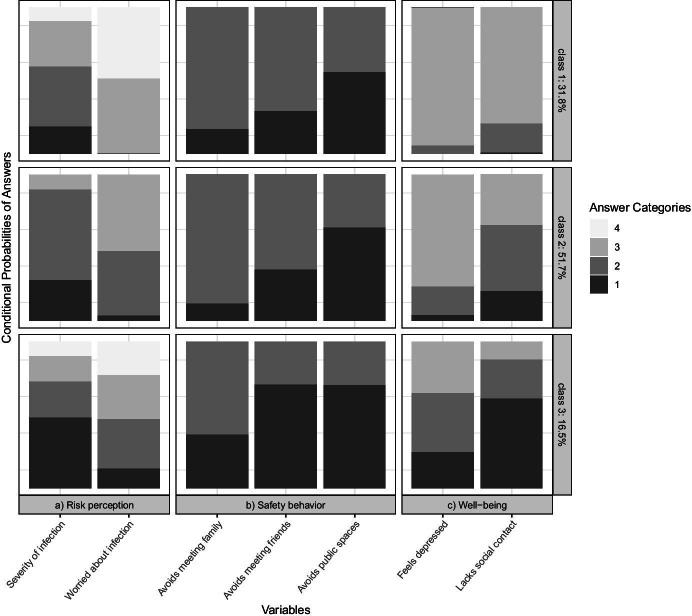
Conditional probabilities for latent class analysis (LCA) model with three latent classes

Our three groups of older German people during the COVID-19 pandemic were interpreted based on the conditional probabilities associated with the manifest variables within each of the three latent classes (see Fig. 1). Answer categories 1, 2, 3, and 4 refer to the categories listed in Table 1. In the case of binary variables, only answer categories 1 and 2 were used. For all variables, a lower value indicates a more profound impact of the COVID-19 pandemic on the different dimensions of older people’s lives.

Class 1 of the LCA included 31.8% of the older people in our sample. This subgroup can be seen as the least affected by the COVID-19 pandemic. The probabilities of answers showed the highest value for most of the answer categories, especially regarding risk perception and well-being. Around half of the *least affected* was not worried at all about getting infected and only a few considered the consequences of an infection as very dangerous. Similarly, almost no one in this group often lacked social contact or had experienced moments of feeling depressed more often since the onset of the COVID-19 pandemic. In terms of safety behavior, the *least affected* showed similarities with those in Class 2. Rather few avoided meeting family members or friends but more than half avoided public spaces.

Class 2 included around half (51.7%) of the older people in our sample. This subgroup can be called the *more affected* as members in this subgroup were more likely to lack social contact, experience moments of feeling depressed, and be strongly or extremely worried about infection compared to their counterparts in Class 1. None of the more affected was not worried at all about an infection and almost everyone considered the consequences of an anticipated infection as very or rather dangerous. Very few avoided meeting family members but more than two-thirds refrained from public spaces. Quite similar to the *least affected*, around one-third of the members of this subgroup avoided meeting friends.

Class 3 was the smallest of the three subgroups, including 16.5% of the older people in our sample. The probabilities of answers showed the lowest value for all answer categories. Therefore, this subgroup can be seen as the *most affected* by the COVID-19 pandemic. Particularly striking was the large share of members lacking social contact and experiencing moments of being depressed often or every now and then since the onset of the COVID-19 pandemic. Although the majority of the most affected met family members, more than half avoided meeting friends and going to public spaces. Regarding risk perception, the most affected were nearly split-half between those being extremely or strongly worried and those being less or not worried at all. The answers were similarly diverse in case of the anticipated consequences of an infection, although those considering them as very dangerous made up for the largest share.

Figure 1 shows a certain similarity between the probabilities of some answers across the three subgroups. When it comes to avoid public spaces, for example, there were no serious differences amongst the members of each subgroup. In addition, the conditional probabilities of some answers seemed to change jointly between the subgroups. According to this, a lower level of risk perception seemed to go hand in hand with better well-being. This echoes findings from previous studies, which found that being worried about the COVID-19 pandemic is associated with lower well-being (Kivi et al. 2021). In some answers, gradual changes in the probabilities could be observed, indicating a linear trend from one subgroup to another. This particularly refers to risk perception and well-being.

### Who belongs to which subgroup?

A more detailed exploration looked into the different subgroups of older people during the Covid-19 pandemic by predicting which of the three latent classes respondents will fall into. In latent class regression models, we employed the following covariates: age, gender, living arrangement, having children, and chronic illness.

**Fig. 2:**
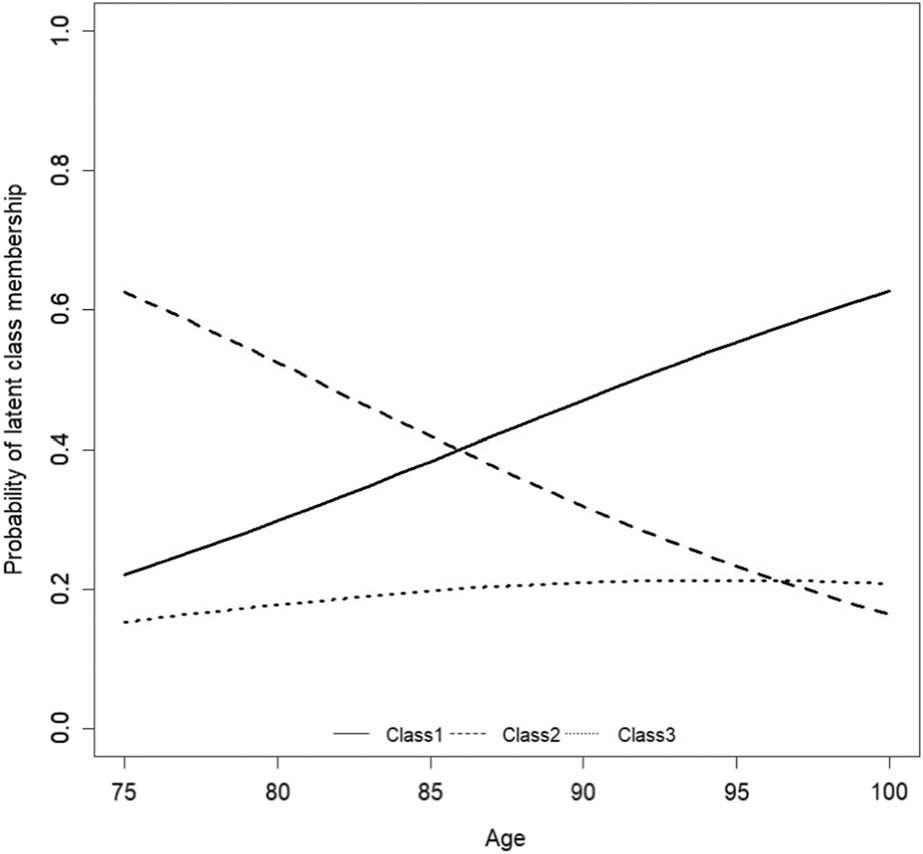
Age as a predictor of subgroup membership

Figure 2 shows the probabilities for falling in the three classes by age. The outcome shows that age correlated with membership in class 1 and class 2, but not with membership in class 3. Hence, the probability to belong to the subgroup of the least affected increased with increasing age, whereas the probability to belong to the subgroup of the more affected decreased with increasing age.

**Fig. 3:**
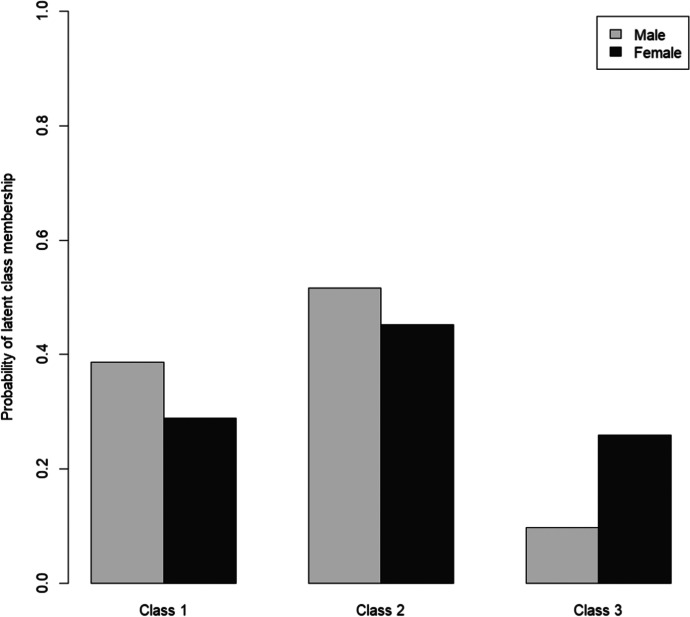
Gender as a predictor of subgroup membership

**Fig. 4:**
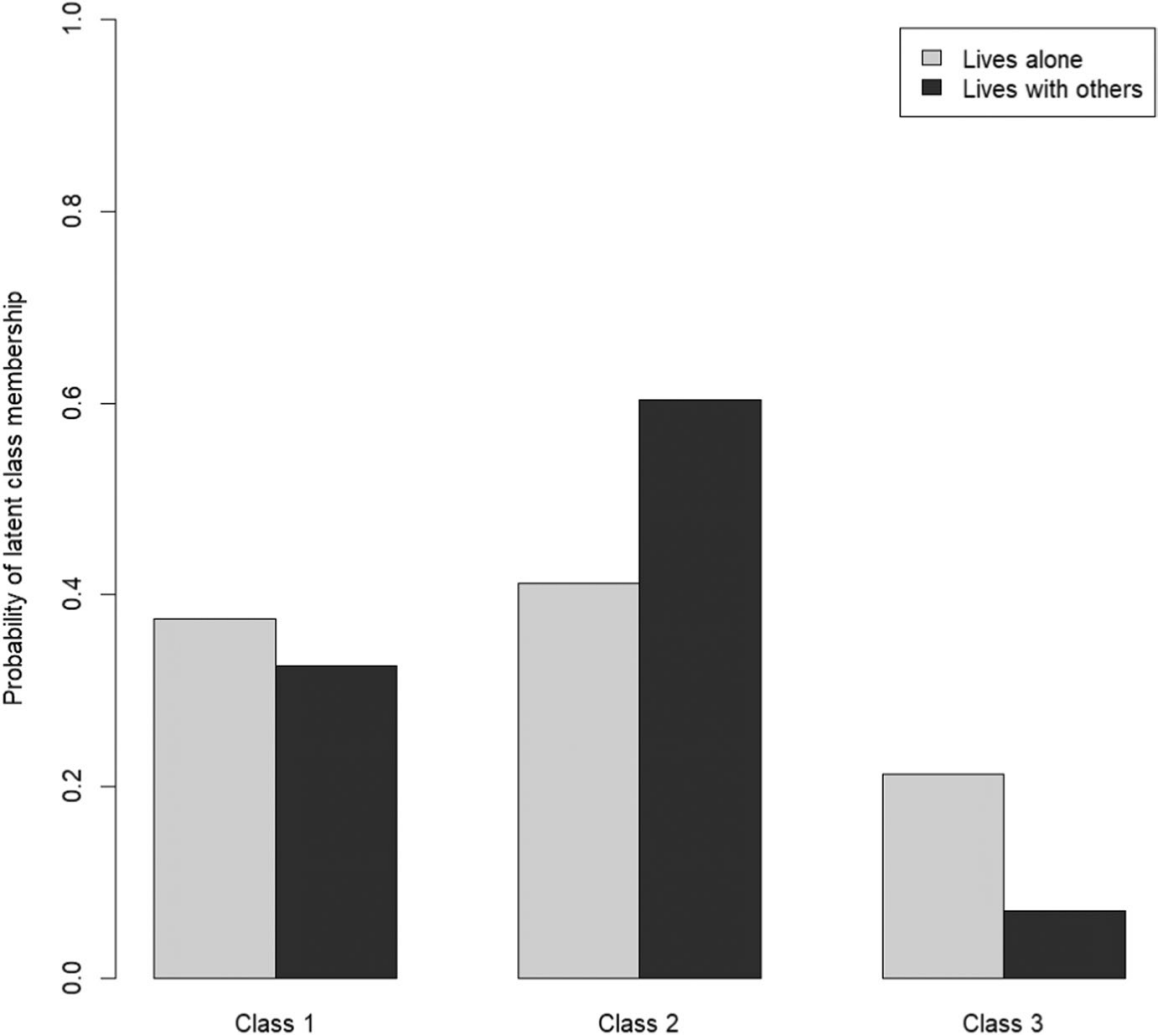
Living arrangement as a predictor of subgroup membership

Figure 3 shows the probabilities for the three classes by gender. Gender differences could be observed in all subgroups, with men having a higher probability to fall in the subgroup of the least or the more affected. The most striking finding, however, was that older women in Germany were around three times as likely to fall in the subgroup of the most affected than older men. Those living alone were also more likely to be found in the subgroup of the least affected. However, living alone also seemed to increase the probability of belonging to the subgroup of the most affected (Fig. 4). Living with a spouse or partner, in contrast, considerably increased the probability of respondents falling in the subgroup of the more affected.

**Fig. 5:**
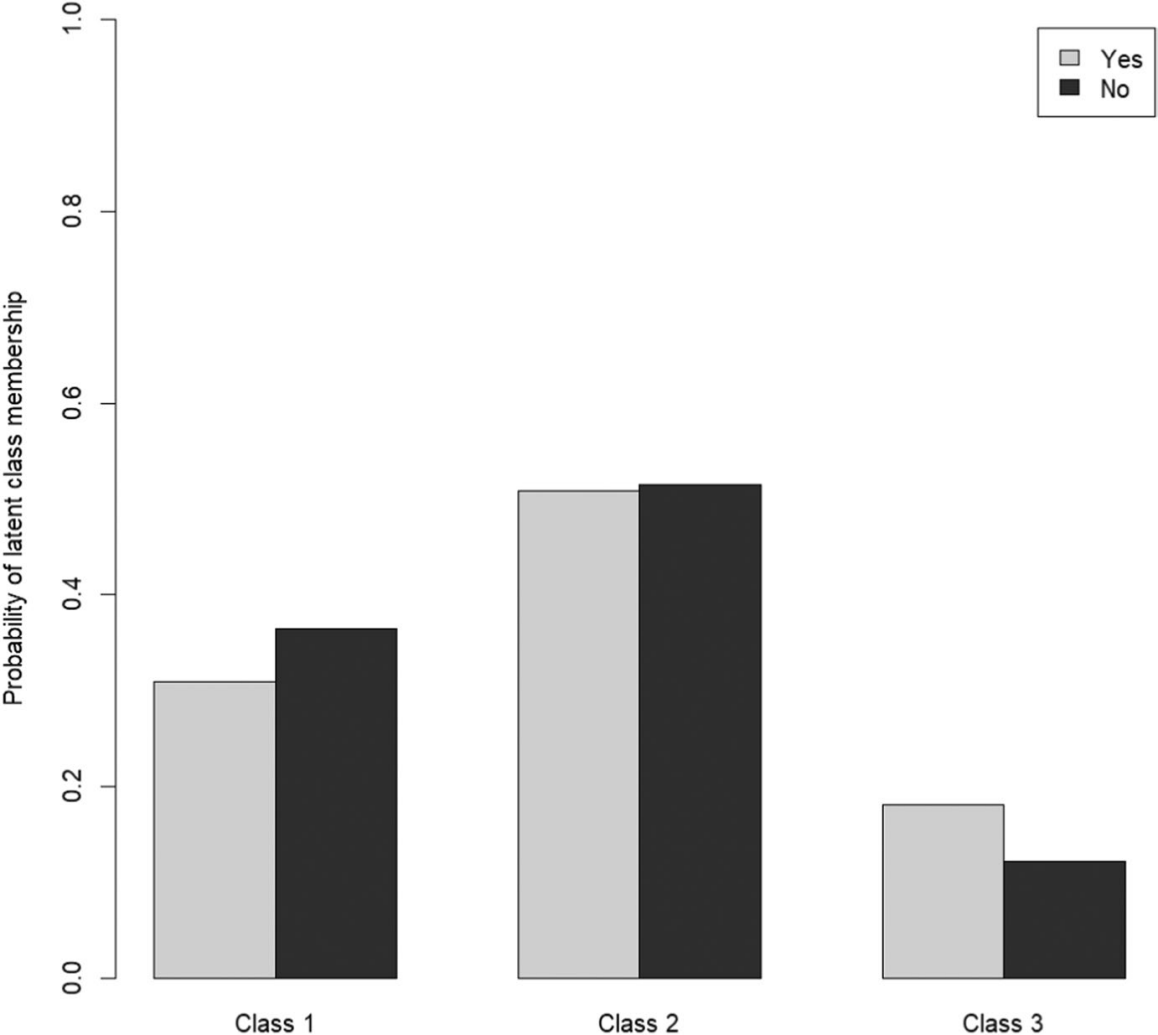
Having children as a predictor of subgroup membership

**Fig. 6:**
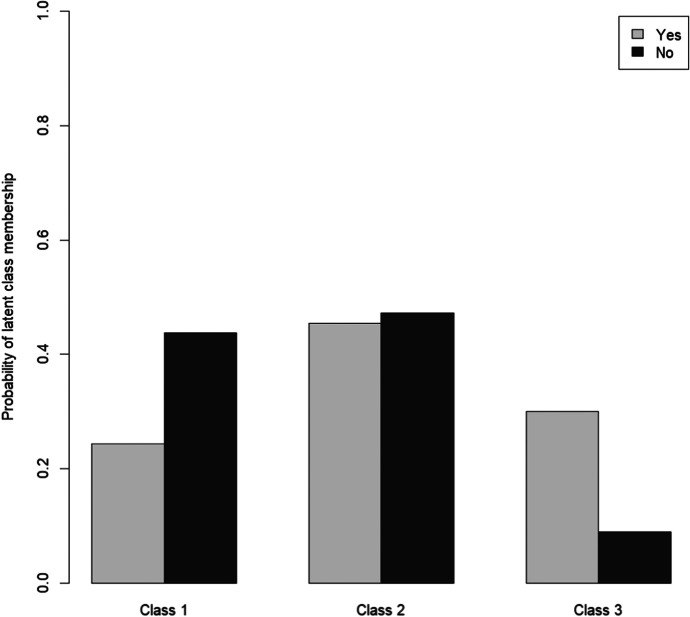
Chronic illness as a predictor of subgroup membership

**Fig. 7:**
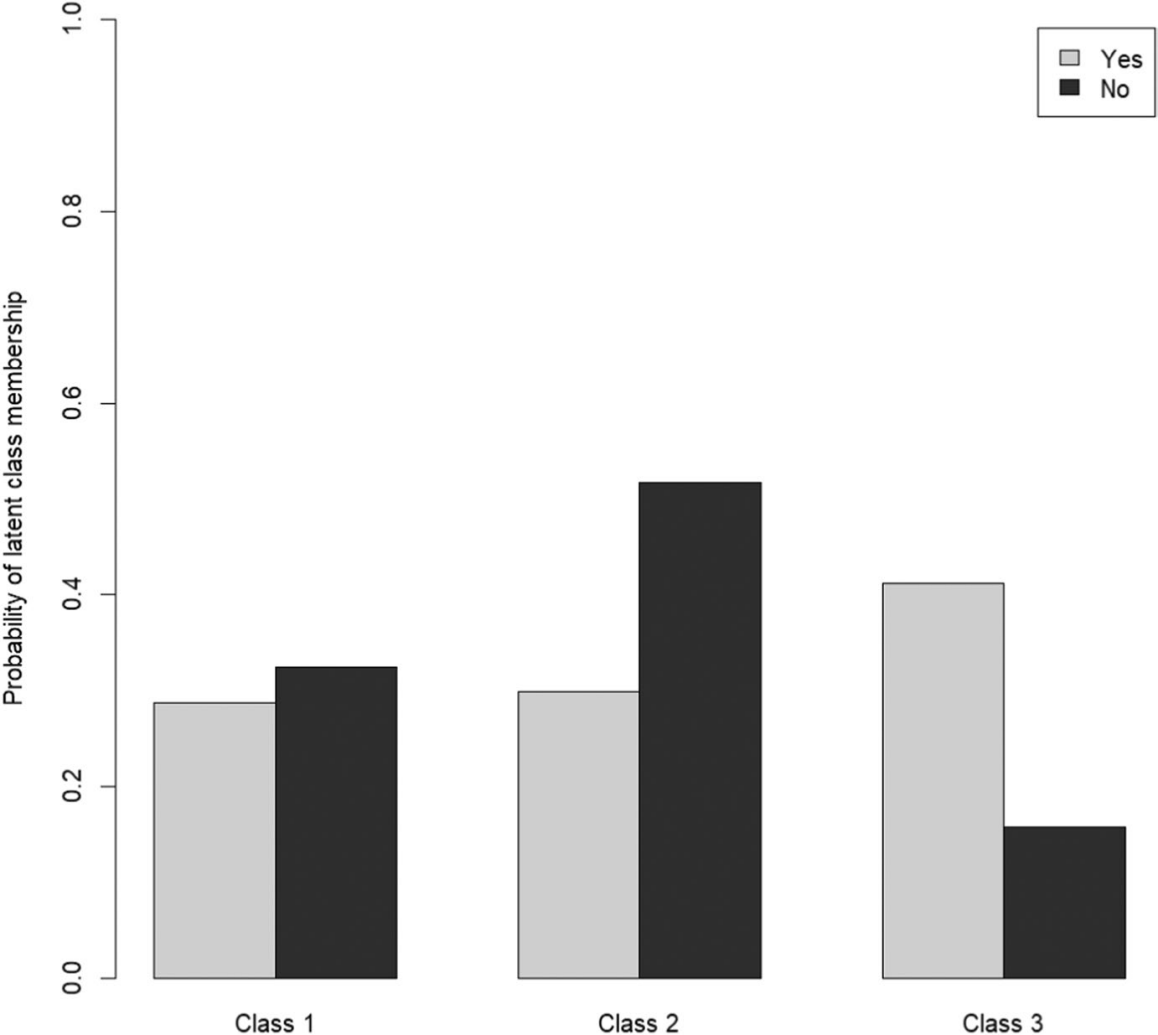
Conflict as a predictor of subgroup membership

According to Fig. 5, having children was not a predictor for membership in the subgroups of the least and the more affected. By contrast, having children seemed to increase the probability to fall in the subgroup of the most affected. Being chronically ill as well seemingly increased the likelihood for membership in the subgroup of the most affected (Fig. 6) as well as reporting more frequent conflicts with family members since the beginning of the COVID-19 pandemic (Fig. 7).

Finally, we controlled for different socioeconomic covariates to identify a possible relationship between education, household income, property and older people’s experiences and perceptions during the COVID-19 pandemic. Interestingly, neither household income nor education or property showed a notable relationship with subgroup membership. Lastly, we controlled for migration history as an indicator for subgroup membership. Again, no notable relationship could be found, although not having a migration history was slightly associated with membership in the subgroup of the most affected.

## Discussion

This study aimed to form different subgroups of older people during the COVID-19 pandemic based on their risk perception, safety behavior and well-being. Findings from LCA indicate that older people can indeed be classified and quantified along with these indicators. With the exception of avoiding meeting family, the probabilities of all indicators change gradually from one group to another, corroborating theoretical assumptions about their interrelatedness. Due to the nature of our data (cross-sectional), no statements about the causal effects between the three main constructs can be made. While risk perception seems to have a bearing on safety behavior (Lu et al., 2021), the link between risk perception and well-being is probably more complex. Thus, older people’s well-being may have an effect on how they perceive the COVID-19 pandemic. At the same time, their well-being can be influenced by their perception and the implementation of safety behavior, such as social distancing.

A finding nurturing the assumption of such a mutual influence is the connection between chronic illness and belonging to the subgroup of the most affected. On the one hand, various studies have shown that chronic illnesses increase the risk of loneliness and depressive moods in older people (Kristensen et al., 2019; Read et al., 2017). Thus, older people particularly affected might have had mental and social health problems already prior to the COVID-19 pandemic (Pierce et al., 2021). On the other hand, being chronically ill has been widely communicated to increase the risk of mortality in the case of a COVID-19 disease significantly (Esme et al., 2021; Parohan et al., 2020). This would explain why members of the subgroup of the most affected are more likely perceiving an infection with Sars-COV-2 as dangerous and rather refrain from meeting family members and friends. The combination of a higher risk of mortality due to chronic illness and a, therefore, higher level of worry and a more profound reduction of personal social interactions is probably the main reason why members of this subgroup experiencing far more severe consequences of the COVID-19 pandemic on their well-being than their counterparts in the other two subgroups.

However, not always may older people have been the ones deciding whether to have personal contact or not. In fact, their personal risk assessment might have differed from the one of their relatives and/or friends. Children, for instance, may have refrained from in-person contact to avoid a possible infection against their older parents’ own wishes. Support for this assumption is found in the higher probability of membership in the subgroup of the most affected in case of more frequent conflicts with family members. This may surprise, as especially children could have been expected to help their older parents to cope with pandemic-related challenges by providing emotional, practical, and personal support. However, as suggested by our results, having children even seems to increase the likelihood of belonging to the subgroup of the most infected. While the result does not rule out the possibility that children play an important role in supporting their older parents in the COVID-19 pandemic, it points to the particularly challenging situation faced by families with older people having a higher risk of infection due to their health status. Nevertheless, the results regarding the effect of having children on membership in the various subgroups should be interpreted with some caution, as the groups of those with and without children in our sample are very unequal in size.

Whereas chronic illness seemed to be a specific predictor for membership in the subgroup of the most affected, age was found to only relate with membership in the subgroups of the least and the more affected. Hence, the younger old were more likely to be found in the subgroup of the more, and the very old were more likely to be found in the subgroup of the least affected. One possible explanation for the fact that the probability of belonging to the group of the least affected increased with increasing age could be that relatively young older people experience a more profound restriction in their social and cultural life than older people at the end of life phases. In addition, in anticipation of increasing health impairments, loss of their partners etc. the relatively young may feel that they have been deprived of important life time. Similar results regarding age were found by Bruine de Bruin (2021) Pasion et al. (2020) exploring risk perception among older people. However, when aiming to detect the particularly affected older people by the COVID-19 pandemic, age did not seem to play a crucial role.

By contrast, gender differences could be observed in all subgroups, with men having a higher probability to fall in the subgroup of the least and the more affected. The most striking finding, however, was that older women in Germany were around three times as likely to fall in the subgroup of the most affected than older men. In this regard, various survey studies had shown, that women respond differently to the COVID-19 pandemic in terms of risk perception, behavior, and psychological distress than men (Barber and Kim 2021; Robbins et al., 2021). This result may seem a bit puzzling in view of the fact that men have a higher risk of a more severe course of the disease and to die because of COVID-19 (Gebhard et al., 2020; Peckham et al., 2020). Approaches to explain gender differences in experiencing the COVID-19 pandemic explained these differences, for example, by learned emotional reactions to threats with women responding with a higher level of fear and anxiety to potential risks than men (Alsharawy et al., 2021; Van der Vegt & Kleinberg, 2020).

Besides sociocultural factors, gender differences may to a certain degree be linked to women’s greater likelihood of living alone in old age. In Germany, for example, 50.4 percent of women between the ages of 74 and 85 live alone, compared to just 21.5 percent of men (Statistisches Bundesamt, 2021). Similarly, in our sample, 59.6 percent of the female respondents reported living alone compared to only 30 percent of their male counterparts. As Fingerman et al. (2021) had shown, older people living alone during the COVID-19 pandemic were less likely to have in-person contact or to receive or provide help than usual. Moreover, compared to those living with others, phone contact with family or friends was associated with higher levels of negative emotions such as loneliness or depression among those living alone. This result is partly supported by the finding of this study that older people living alone rather fall into the group of the most affected. However, those living alone also tended to rather fall into the group of the least affected. As previously shown, the probability to be a member of this subgroup increased with increasing age. At the same time, the likelihood of living alone increased with each year of life after the age of 75, for both older women and men in Germany (Statistisches Bundesamt, 2021). This means that at least a part of the least affected were very old people living alone. In other words, living alone was not per se a risk factor for older people during the COVID-19 pandemic.

Surprisingly, no evidence was found for an association between subgroup membership and socioeconomic status measured in terms of education, household income, and property. According to fundamental cause theory (Link & Phelan, 1995), it might have been expected that the COVID-19 pandemic would put older people with fewer socioeconomic resources at a higher risk of reduced well-being (Wachtler & Hoebel, 2020). Some studies indeed found correlations between a lower socioeconomic status and a greater risk for psychological distress among older people (Sams et al., 2021). By contrast, investigating the impact of policy measures for social distancing on the well-being of older people, Van Tilburg et al. (2020) did not find a correlation between socioeconomic variables, loneliness, and mental health. Although results from these studies are difficult to compare because of methodological reasons (e.g., measurements, type of data), socioeconomic status may not be appropriate as the sole explanation for well-being outcomes during the COVID-19 pandemic (Wanberg et al., 2020). Therefore, more attention should be paid to the linkages between different resources such as household income, knowledge about COVID-19, resources for resilience, family support, etc.

Similarly, the finding that older people with a migration history are not more likely to belong to the group of the most affected may seem surprising. Studies from the United States have shown that members of ethnic minorities are exposed to a higher COVID-19 infection and mortality risk than members of the majority society (Millett et al., 2020; Rodriguez-Diaz et al., 2020; Sáenz and Garcia, 2021). These findings have been explained by longstanding ethnic health inequalities among older people, deeply rooted in structural racism within American society (Garcia et al., 2021). Although people with a migration history tend to be exposed to a relatively higher risk of COVID-19 infection and mortality in many European countries as well (Hayward et al., 2021; Indseth et al., 2021), there is still very scant research specifically on older people from ethnic minorities. A possible explanation for this study not finding differences may be the small sample of older people with a migration history (only ten percent of all respondents fall into this category). Alternatively, older people with a migration history may have distinct strategies to cope with COVID-19 related stress, unobservable in our data.

## Conclusion

This study aimed to classify and study different subgroups of German older people during the COVID-19 pandemic. It showed that three subgroups of German older people can be formed based on their risk perception, safety behavior, and well-being. Results from LCA reveal that the likelihood of higher risk perception, adherence to safety behavior, and reduced well-being increases from one subgroup to another. The findings indicate the usefulness of these interrelated concepts for identifying and quantifying older people particularly affected by the COVID-19 pandemic and the measures taken to contain it. According to this, around 16 percent of German older people are faring relatively worse with gender (being female), health (chronic illness), children (having children), and living arrangement (living alone) predicting membership to this subgroup. In view of the predictors identified, it can be assumed that these older people already belonged to a particularly vulnerable group due to their health conditions and social situation before the outbreak of the COVID-19 pandemic.

As far as social interventions are concerned, older people belonging to the subgroup of the most affected should be specifically targeted by social support programs in their immediate environments. A particular challenge when direct social contact should be avoided. Therefore, besides the detection or knowledge of need, the establishment of trustful relationships despite social distancing is crucial. Phone calls (e.g., “window-talking”), letters, or little presents could contribute improving older people’s well-being, even beyond the COVID-19 pandemic. As having children and more frequent conflicts with family members predict membership to this subgroup, social interventions should not only focus on older people but their families as well. How, e.g., can families be supported when care for older people is difficult because of social distancing? How can communication between older people and their family members be facilitated when visits are not possible?

At the same time, our results show that many older people have coping resources that enable them to deal with the consequences of the COVID-19 pandemic. These older people have managed to adapt to the new situation and to face the challenges it poses for their everyday and social life. This is especially true for the very old, who, in contrast to the younger old, are most likely to be found in the subgroup of the least affected. Although this finding goes in line with results from previous studies, the underlying mechanisms of this finding are still unclear. Qualitative rather than survey study designs might help shed light on this phenomenon by exploring the complex relationships between stages in the life course, risk perception, resilience, and well-being during the COVID-19 pandemic.

Moreover, future research may analyze longitudinal data using LCA in order to explore causal relationships as well as changes in subgroups of older people over time. Such an analysis would allow us to relate these changes to the course of the pandemic and the measures taken to lower the number of deaths and infections. More in-depth analysis on the role of socioeconomic status and its association with other factors (e.g., knowledge about COVID-19, coping strategies) is needed as well. Similarly, more research is needed on differences and similarities between ethnic minorities and members of the majority society, but also on differences and similarities between and within ethnic minority groups.

This study has different limitations. First, it is based on data gathered at a specific moment during a very dynamic process. Thus, at the time of data collection Germany had passed through a relatively mild first wave of the pandemic and the extent of the second wave, which should be significantly larger, could not yet be anticipated. Second, it has to be considered that the sample consisted exclusively of older people who lived in their own homes. Compared to older people who are accommodated in nursing homes, they are exposed to a significantly lower risk of being infected by nursing staff or visitors. At the same time, those living at home were not affected by various months of bans of receiving visitors, as were those living in nursing homes or those having to stay at hospital.

Finally, the variables included as indicators in the LCA model may eventually be criticized for not adequately measuring the three central constructs (risk perception, safety behavior, and well-being). This criticism is right in that none of the constructs has been operationalized by standardized measurements. Nevertheless, the relationship between subgroup membership and covariates goes in line with findings from previous studies and thus indicates the reliability of our measurements. Further concerns might be levelled against the replicability of our findings. However, by randomly splitting our data set we found class probabilities to be relatively stable, suggesting the applicability of our model to other data sets.

## Supplementary Information

Below is the link to the electronic supplementary material.

Supplementary file1 (DOCX 15 KB)

Supplementary file2 (DOCX 210 KB)

## Data Availability

The R Script is available as part of the supplementary material. Access to the data will be provided to researchers showing a serious interest.
