# Supplementary material for: Older People in Germany During the COVID-19 Pandemic:The Least, the More, and the Most Affected
Source: J Popul Ageing. 2021 Dec 13;16(1):5–26. doi: 10.1007/s12062-021-09352-4 (PMC8666192; doi:10.1007/s12062-021-09352-4)
Supplement: Supplementary file 1 — Supplementary file1 (DOCX 15 KB) [file 12062_2021_9352_MOESM1_ESM.docx]

| **Variables** | **1** | **2** | **3** | **4** | **5** | **6** | **7** | **8** | **9** | **10** | **11** | **12** | **13** | **14** | **15** |
| --- | --- | --- | --- | --- | --- | --- | --- | --- | --- | --- | --- | --- | --- | --- | --- |
| **1 Worried about infection** | 1 |  |  |  |  |  |  |  |  |  |  |  |  |  |  |
| **2 Severity of infection** | 0.24*** | 1 |  |  |  |  |  |  |  |  |  |  |  |  |  |
| **3 Avoids meeting family** | -0.02 | -0.07 | 1 |  |  |  |  |  |  |  |  |  |  |  |  |
| **4 Avoids meeting friends** | 0.08 | 0.05 | 0.20*** | 1 |  |  |  |  |  |  |  |  |  |  |  |
| **5 Avoids public spaces** | 0.07 | 0.06 | 0.13** | 0.18*** | 1 |  |  |  |  |  |  |  |  |  |  |
| **6 Lacks social contact** | 0.26*** | 0.07 | 0.03 | 0.12** | 0.02 | 1 |  |  |  |  |  |  |  |  |  |
| **7 Feels depressed** | 0.19*** | 0.10* | 0.06 | 0.09 | 0.00 | 0.32*** | 1 |  |  |  |  |  |  |  |  |
| **8 Age** | 0.09 | 0.02 | 0.01 | -0.02 | -0.09* | 0.07 | -0.02 | 1 |  |  |  |  |  |  |  |
| **9 Child(ren)** | -0.01 | -0.03 | -0.18*** | 0.03 | -0.05 | 0.01 | 0.04 | 0.02 | 1 |  |  |  |  |  |  |
| **10 Gender** | 0.01 | -0.07 | -0.01 | 0.00 | -0.12** | -0.11* | -0.19*** | -0.01 | -0.01 | 1 |  |  |  |  |  |
| **11 Living arrangement** | 0.04 | -0.02 | -0.12** | -0.08 | -0.02 | -0.01 | -0.03 | 0.06 | 0.07 | 0.09 | 1 |  |  |  |  |
| **12 Chronic Illness** | 0.12** | 0.11* | 0.06 | 0.01 | -0.03 | 0.12** | 0.17*** | 0.01 | 0.04 | -0.11* | -0.12** | 1 |  |  |  |
| **13 Conflict** | 0.05 | -0.03 | 0.00 | 0.00 | -0.06 | 0.11* | 0.12** | 0.03 | 0.05 | 0.01 | 0.01 | 0.17*** | 1 |  |  |
| **14 Migration** | 0.02 | -0.04 | 0.01 | 0.03 | 0.02 | -0.05 | -0.03 | 0.02 | 0.07 | -0.06 | -0.04 | -0.01 | -0.04 | 1 |  |
| **15 Education** | -0.01 | 0.00 | -0.07 | -0.11* | -0.13** | 0.05 | 0.04 | 0.03 | 0.02 | 0.20*** | 0.03 | -0.01 | -0.04 | 0.00 | 1 |

Table 2. Bivariate Correlation Matrix

Spearman’s correlation coefficient; *p < 0.5, **p < 0.01, ***p < 0.001e
