# Supplementary material for: Older People in Germany During the COVID-19 Pandemic:The Least, the More, and the Most Affected
Source: J Popul Ageing. 2021 Dec 13;16(1):5–26. doi: 10.1007/s12062-021-09352-4 (PMC8666192; doi:10.1007/s12062-021-09352-4)
Supplement: Supplementary file 2 — Supplementary file2 (DOCX 210 KB) [file 12062_2021_9352_MOESM2_ESM.docx]

Supplementary material: Goodness of fit measures for latent class analysis (LCA) models


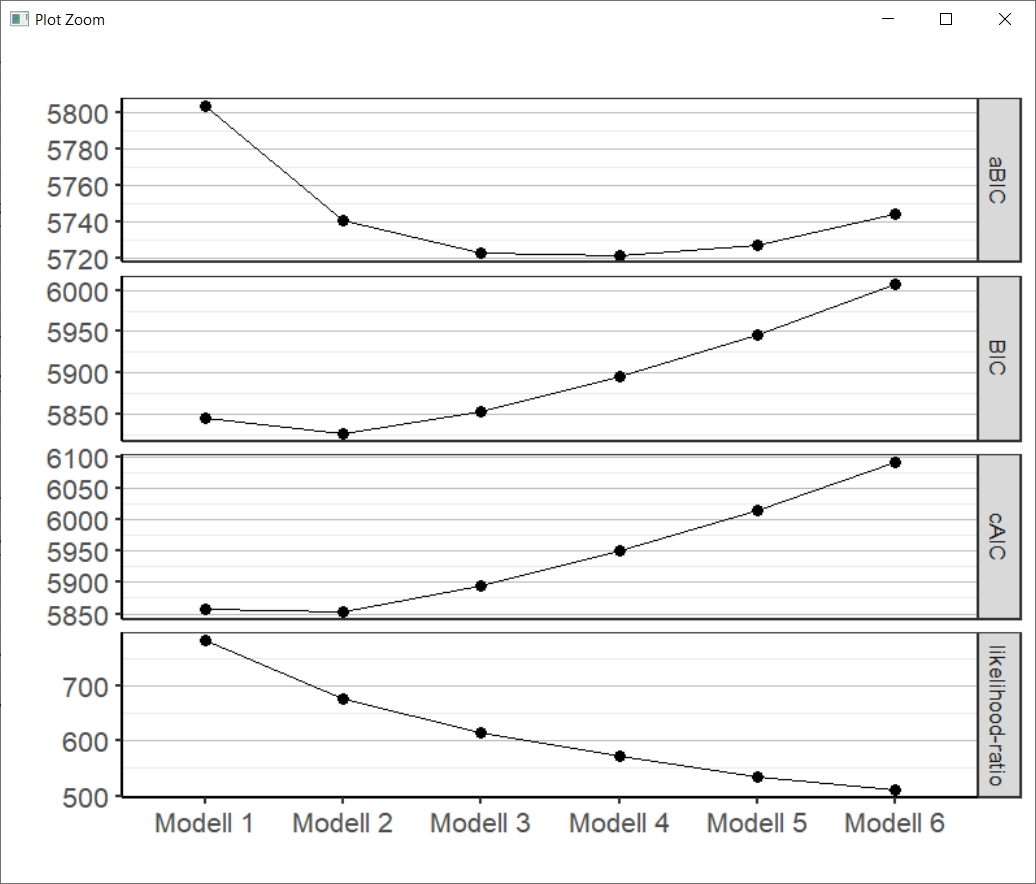


| Model | log-likelihood | resid. df | BIC | aBIC | cAIC | likelihood-ratio | Entropy |
| --- | --- | --- | --- | --- | --- | --- | --- |
| Modell 1 | -2882.37 | 479 | 5845.31 | 5804.05 | 5858.31 | 781.71 | - |
| Modell 2 | -2829.38 | 465 | 5826.13 | 5740.43 | 5853.13 | 675.75 | 0.578 |
| Modell 3 | -2799.32 | 451 | 5852.78 | 5722.65 | 5893.78 | 615.62 | 0.63 |
| Modell 4 | -2777.58 | 437 | 5896.08 | 5721.51 | 5951.08 | 572.14 | 0.596 |
| Modell 5 | -2759.11 | 423 | 5945.92 | 5726.91 | 6014.92 | 535.2 | 0.672 |
| Modell 6 | -2746.72 | 409 | 6007.91 | 5744.47 | 6090.91 | 510.41 | 0.642 |
